# Performance evaluation and financial viability analysis of grid associated 10 MW_P_ solar photovoltaic power plant at UP India

**DOI:** 10.1038/s41598-022-26817-4

**Published:** 2022-12-26

**Authors:** Shailendra Singh, Majed Alharthi, Abhishek Anand, Amritanshu Shukla, Atul Sharma, Hitesh Panchal

**Affiliations:** 1 Non-Conventional Energy Laboratory, Rajiv Gandhi Institute of Petroleum Technology, Jais, Amethi India; 2 Department of Physics, University of Lucknow, Lucknow, India; 3 Mechanical Engineering Department, Government Engineering College Patan, Patan, Gujarat India; 4 Finance Department, College of Business, King Abdulaziz University, P.O.BOX. 344, Rabigh, 21911 Saudi Arabia

**Keywords:** Energy science and technology, Engineering

## Abstract

The main aim of this simulation work is to assess the financial possibility analysis of 10 MW_P_ grid-associated solar photovoltaic (PV) power plants in seven cities i.e. Lucknow, Agra, Meerut, Gorakhpur, Kanpur, Allahabad, and Varanasi of Uttar Pradesh (UP) state of India with the RETScreen Software. The presented research work demonstrates the method of selection of profitable locations for solar PV power plants according to financial viability indicators. It is found that Allahabad city is the most profitable site with values of 16,686 MWh of electricity exported to the grid (EEG), US$20,896.30/year of electricity export revenue (EER), 9.4 years of simple payback period (SPP), 7.7 years of equity payback period (EPP), 19545.9 tCO_2_/year of GHG emission reduction, US$3492.82/year of the annual life cycle savings (ALCS), 1.5 benefit-cost (B-C) ratio, US$27394.59 of net present value (NPV), 16.5% internal rate of return on equity (IRR-equity), 12.3% modified internal rate of return on equity (MIRR-equity), 5.4% internal rate of return on assets (IRR-assets), and 7% modified internal rate of return on assets (MIRR-assets). The second most profitable site is found in Gorakhpur city and Varanasi city is found the least profitable site. The other two cities, Agra and Kanpur are not found suitable because of the negative values of NPV and ALCS.

## Introduction

The electricity generated from the combustion of fossil fuel, i.e. coal-based thermal power plant (CBTPP), is the main cause of greenhouse gas (GHG) emissions and air-polluting agents like sulfur oxide (SO_x_), nitrogen oxides (NO_x_), carbon oxides (CO_x_), suspended particulate matter (SPM), lead, non-methane hydrocarbons, mercury, etc. India has a major share of environmental pollution due to CBTPP electricity generation i.e. for the year 2016, the power sector reports the total environmental pollution for SO_2_ of 51%, CO_2_ of 43%, NO_x_ of 20%, and SPM of 7%. The entire connected capacity of the CBTPP of India was 365 GW as of 31 March 2019 and these plants are generating about 75% of the electricity of the country^1^. According to the observation of the air pollution of 2.5 SPM, out of the ten most populated cities around the world, nine cities are located in India i.e. Kanpur, Faridabad, Gaya, Delhi, Gurugram, Varanasi, Agra, Lucknow, and Patna. In the present century, the prevalent challenge is to balance our environment and ecological system along with the economic development activities of humans. It has been observed that approximately 1.0 °C of global warming is occurred due to only the developing activities of humans. It is expected that if global warming continues at the same rate then it will reach 1.5 °C between 2030 and 2052^2^. There is a necessary shifting of the mechanism of electricity generation from fossil fuel-based to sustainable energy-based like solar energy to curb the disastrous impact of global warming and air pollution in the present era.

There are mainly three routes for the mitigation of climate change i.e. conventional mitigation technologies (CMTs), negative emission technologies (NETs), and radiative forcing geoengineering (RFG). CMTs target reducing fossil-based CO_2_ emissions. NETs capture and sequester atmospheric carbon to reduce the CO_2_ level. RFG technique modifies the earth's radiative energy budget to lower or stabilize the global temperature. At the 21st United Nations Climate Change Conference held in Glasgow, the respective countries agreed to work together to combat the negative impact of climate change and to keep global warming below 2 °C with a target of 1.5 °C. At the 26th meeting in Glasgow, new commitments were made to reduce carbon emissions. The progress was made in four key areas i.e. coal, cars, cash, and trees. It was agreed to phase out coal which is the most polluting fuel, replacement of fuel-based vehicles with electric vehicles, and $100 billion in financing to the developing countries. The four major outcomes of this conference were to secure a global net zero by mid-century and keep 1.5 °C within reach, adapt to protect communities and natural habitats, mobilize finance, and work together to deliver. It was agreed that developed countries and ones with large carbon emissions would take the lead, the government would help the most vulnerable, the government should promote greener and climate-resilient infrastructure and support technological innovations, and everyone should work together and there would be cooperation among governments, national functional sectors, and financial institutions^3^.

According to the 19th electricity power survey of India, it is expected that the collective electricity demand could be grown between 2074 and 2785 TWh by the year 2030^4^. This demand could not be fulfilled by fossil fuels alone due to the inadequate quantity of them and the adverse impact of global warming on climate. To meet this electrical energy demand, the solar energy-based sustainable technology of power generation might be a credible solution for developing countries like India. India has committed to achieving 40% of aggregate electricity installed from non-conventional sources of energy like solar energy. It is expected that it accounts for the total power generation capacity of 18% from 1% at present. It is showing the main role of the nation’s attempt to attain the renewable energy target of 350 GW by the year 2030^5^.

The UP state of northern India is located at 26.85°N and 80.91°E and has a humid subtropical climate. The state receives an excellent quantity of sunlight all over the year, it has average annual solar energy of 4.6 kWh/m^2^/day^5^. The installed capacity of power generation of the state has grown from 1362 MW in the year 1947 to 22,602 MW in the year 2017^6^, which shows the rapid urbanization and socio-economic growth of the state. It had been recorded that the state was generating about 22,602 MW of electricity in the year 2017 which was 7% of the entire fitted capacity of the country, but a major share of 80% of this generation was coming from a coal-based technology^7^. The Ministry of New and Renewable Energy (MNRE), Government of India has set targets to meet the demand and supply of electricity for urban as well as rural households for twenty-four hours, through the solar energy potential of 22,300 MW capacities. To achieve this target, the state should have to generate solar energy power of 10,700 MW capacity.

In the present study, the financial viability of seven cities has been done for the state of UP of India for 10MW_p_ solar PV power plants to fulfill the electrical needs of the state. This type of simulation study is very important for developing countries like India, Pakistan, Bhutan, Nepal, the Philippines, Sri Lanka, etc. Because the study has the potential to exhibit the application of solar energy resources for specific regions. Through the study, financial parameters can be investigated in detail. With help of these parameters, a decision for installing solar PV power plants at the most profitable site can be taken place. Apart from the financial parameters, the study also explains the environmental benefits in the form of mitigation of harmful GHG gases.

The authors went through some prominent simulation work related to our simulation modeling of solar PV power plants like Harder and Gibson^8^ investigated the production of energy, financial possibility, and decreases of GHG emission for 10 MW_P_ solar PV power plants in Abu Dhabi by RETScreen software. The authors found that estimated savings for CO_2_ of 10,732 tons (equivalent to NO_x_ of 372.8 tons), SO_2_ of 0.15 tons_,_ and total annual SPM of 1.7 tons by switching the yearly production of 24.4GWh from the coal-based power plant to 10 MW_P_ solar PV power plants. Markam and Sudhakar^9^ analyzed the tilt angle of a PV array at six different locations in India for optimal performance through various simulation software like RETScreen, PV-Syst, and NRELSAM. The author's found that the optimal tilt angle at each location is very much near the latitude angle of the location. They observed the optimal tilt angle is varied in the range of + 2 to + 3° from their latitude angle of location. Their analysis also showed that the average value of solar energy at each location was higher than the value of 5 kWh/m^2^/day and fluctuates from 5 to 6.5kWh/m^2^/day. Salehin et al.^10^ analyzed the potential of renewable energy for the generation of electrical power at Kutubdia Island in Bangladesh. Their study included various parameters like financial analysis, cost analysis, and GHG emission analysis for various power generation technologies like solar PV-diesel energy systems and wind-diesel energy systems with the help of HOMER and RETScreen software. Lee^11^ made a simulation model for analyzing the economic viability and policy framework for the PV system at the cohousing in Korea. The author made his model through the RETScreen software. The model included the economic feasibility of the PV power system of a single apartment in Seoul for replacing the existing electrical power system in six major cities of Korea by the year 2030. Trindade and Corderio^12^ made a simulation model for checking and verifying of design for the performance of a stand-alone solar PV system by taking five case studies. The author’s study also includes an analysis of a solar panel, charge controller, battery, inverter, and electric load. Their results show the method of validation for the proposed renewable energy project before buying the equipment. Owolabi et al.^13^ investigated the viability of installing a 6 MW_P_ grid-associated solar PV power plant at six different northern locations in Nigeria. Their model presents the study of technical, financial, sensitivity, risk, monetary, and environmental aspects of setting up a solar power plant system through the RETSceen software. Among the six sites, the authors found that Yobe state was the highest profitable site that contained maximum yearly solar energy of 5.96 kWh/m^2^/day, a maximum capacity factor of 21.7%, the maximum value of energy exported to the grid 11,385 MWh, the least period of payback of 13.6 years, the maximum potential of reduction of GHG emission 5452.5 tons (comparable to 501.5 hectares of carbon-absorbing by the forest), and the highest value of clear sunny days of the state. Bakhshi-Jafarabadi et al.^14^ investigated the monetary possibility of a 120 KW_P_ grid-associated solar PV system for commercial application by using RETScreen clean energy management software in Kashmar, Khorasan Razavi, and Iran of the Middle East region. The author studied a model for a feed-in tariff scheme. They used several economic indices like NPV, IRR, B-C ratio, SPP, and the Levelized cost of energy for determining the performance of the project. Their study revealed that the computed outputs for the commercial system were associated with a 3.36 B-C ratio, IRR of 31.88%, SPP of 5.24 years, and 0.0477 $ per kWh of Levelized cost for energy were very attractive. Kassem et al.^15^ investigated the twenty-two sites of Libya for a 10 MW solar PV power plant for utilization of the solar energy potential of this region. They made a simulation study of all selected locations by making a model in the RETScreen software tool. Their study suggested only two profitable locations among twenty-two locations i.e. Al Kufrah and Murzuq. M.M. Rafique and H.M.S Bahaidarah^16^ analyzed ten cities of Pakistan for a 5 MW solar PV power plant. They found all the selected sites were financially feasible and beneficial in the decrease of GHG emissions, along with Quetta being the most profitable site. They evaluated all the financial and environmental parameters through the RETScreen simulation software. H.K. Jobair and J.M. Mahdi^17^ had investigated a 10 MW solar PV power plant in one city of Iraq namely AI-Anbar, for a sun tracking system. They found that the dual-axis system was more effective for the economic feasibility of the power plant along with the environmental benefits. They did their study with the help of the RETScreen software tool. M.M.Omrani et al.^18^ presented the RETScreen simulation study of a one-axis tracking system for a 10 MW solar PV power plant in ten cities in the southeastern region of Iran. Their analysis suggested that the Port of Jask was the most profitable site among the selected cities. Y. Kassem and H. Gokcekus^19^ investigated the viability of a 1 MW grid-associated solar PV power plant at Lefke town of Northern Cyprus. They did their study through the PVGIS and RETScreen simulation tools. They analyzed various tracking systems like open surface, vertical axis, and two-axis systems. They found that the installation of the PV power plant was advantageous. H. O. Njoku and O. M. Omeke^20^ presented a study of 100 MW solar PV power plants at twenty-five locations in Nigeria with the help of the RETScreen software tool. Their study suggested that the high latitude location of Nigeria was more suitable for the installation of solar PV power plants i.e. Gusa. S. Bentouba et al.^21^ investigated the performance of solar PV power plants for the hot climatic region through the real performance data of the Adrar power plant of southern Algeria. They presented their simulation model for comparing results for two simulation software, Homer Pro and RETScreen Expert. Their study suggested that thin film technology was more beneficial for hot climatic regions. H. Chowdhury et al.^22^ presented a study for the mitigation of GHG emissions produced by airports in Bangladesh. They simulated the proposed model of 5 MW of solar PV power plants at buffer areas in two targeted airports through the RETScreen software tool. They concluded that the proposed model is feasible and viable at the selected sites. S. Sreenath et al.^23^ presented the 7E analysis of 5 MW solar PV power plants at seven airports in India. They made their simulation model through the RETScreen software. They found through their analysis that solar PV power plants were feasible and viable at the selected airports for zero carbon emissions. R. S. Yendaluru et al.^24^ presented the simulation study for hybrid power plant generation. They suggested the solar PV power plant incorporated in the shadow-free space of wind farm power generation. They found power generation capacity was improved by 90% along with 7 years payback period. S. Sreenath et al.^25^ presented a 7E analysis of a 5 MW_P_ solar PV power plant at five locations in Malaysia. They found through their analysis that site 2 is the most suitable site for the allocation of the power plant. A. K. Saxena et al.^26^ examined 100 kW_P_ rooftop-based solar PV power plants in seven cities in India. They had done a 3E analysis of their simulated model made through PVsyst software. They found that all selected cities were feasible for the rooftop-based solar PV power plants. Gopi et al.^27^ analyzed the impact of weather on the performance of solar PV power plants in tropical regions. They did their study by making a mathematical model through the Minitab statistical tool. They found that energy generation has been affected in the rainy season in the humid tropical region. A.K. Saxena et al.^28^ evaluated the 100 kW_P_ solar rooftop-based power plant situated in Bhopal city of India. They analyzed the solar PV system for performance analysis and its associated losses. They made their study model with the help of three simulation software i.e. PVsyst, PVGis, and Solar Gis. Their comparative results of the simulated study are valuable for commercial applications. B. Shiva Kumar and K. Sudhakar^29^ examined in detail the performance of a 10 MW_P_ grid-connected solar PV power plant situated in Ramagundam, India. They analyzed the performance of the power plant with the simulated model made through the software of PVsyst and Solar Gis. They found that their simulated model was in good agreement with the actual performance parameters. S. Sreenath et al.^30^ presented a study of reducing environmental pollution at ten airports in India. They suggested grid-connected solar PV power plants for electricity generation. They analyzed the energy generation through the PV watts calculator.

The authors could not find any specific regional study related to the UP state of India. Which could define the solar resource potential and viability of solar resource applications for electricity generation. So, this study attempts to evaluate the environmental, technical, and financial viability in detail of 10 MW_P_ grid-associated solar photovoltaic power plants at various geographical locations in the UP state for fulfilling the electricity demand. The study analyzes the solar energy potential of various selected sites and through this energy potential, all selected sites are examined according to the financial parameters of viability. This paper also depicts the environmental benefits in terms of the total decrease in yearly GHG release potential. The study presents the method of feasibility for the selection of profitable sites for installing solar PV power plants. The authors expected that the key findings and results of financial indices could be of great interest for deriving the future expansion of solar power plants and the solar policies of the UP state of India as well as for similar climatic regions around the world.

## The solar PV system

**Figure 1:**
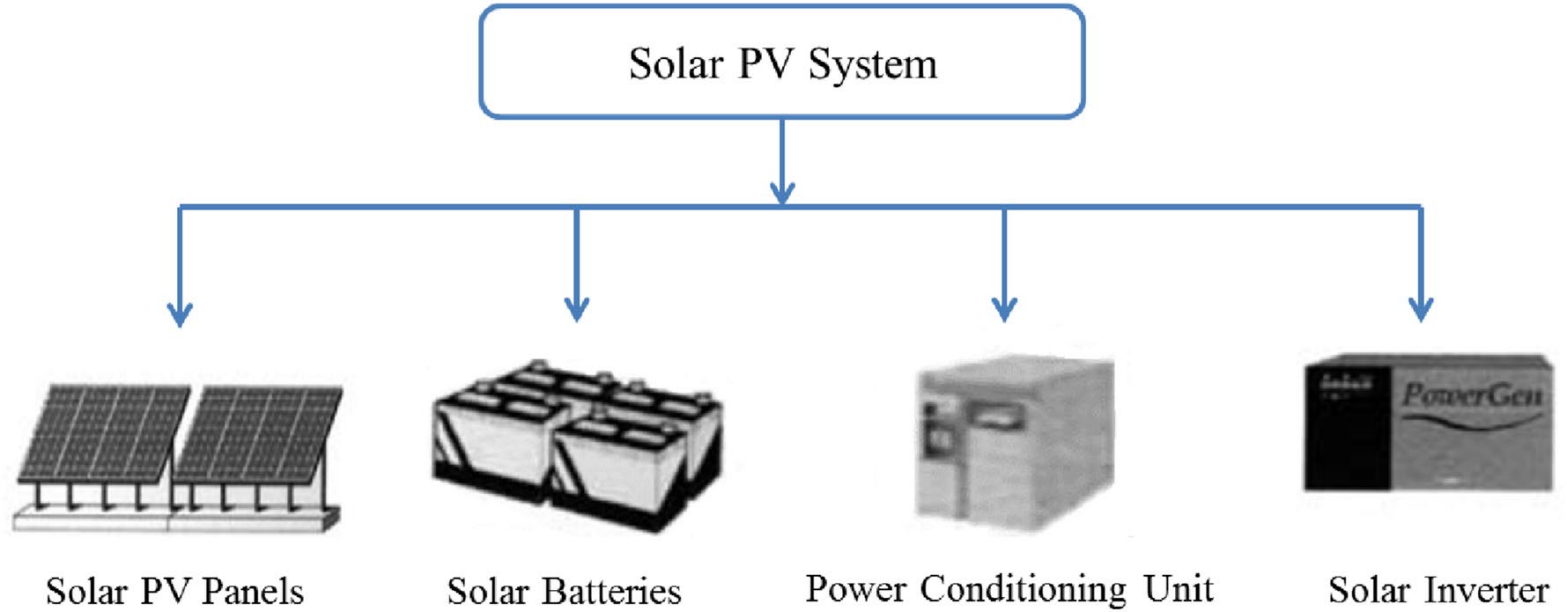
Solar PV components.

The main constituents of a solar PV system are solar PV panels, solar batteries, power conditioning unit, and solar inverter as shown in Fig. 1. These components are chosen according to the requirement of the project and its site selection.

### Solar PV panels

The most essential component of the photovoltaic system is solar panels. It transforms solar energy into direct current with the help of solar cells. Each solar panel contains thirty-six solar cells that are coupled in a series and captured in glass and plastic sets to protect them from the atmosphere. These packages are further connected by an electrical connection to the junction box. The efficiency range of the crystalline module is found from 11 to 15% for the transformation of solar energy into electrical energy and the average life of modules are ranging from 25 to 30 years. In this sequence, one of the key aspects of the PV modules is the accumulation of PV waste due to their short life span i.e. 25–30 years. The increase in the installations will lead to the accumulation of a large amount of PV waste in the near future^31^. The PV modules usually end up in landfills with other municipal solid wastes (MSW) or incinerated with little emission control leading to the release of harmful and carcinogenic substances into the atmosphere. The pyrolysis process can contribute positively to the recycling rates by processing the waste polymers into potential fuel sources and thus contribute to the circular economy. The PV modules contain very precious and rare metals that are obtained during the mining of the earth viz. Ag, Cu, Al, Sn, and Pb. The reserves of these metals will only last a few years with the current rate of extraction. There is great potential in terms of value lying in the end-of-life (EoL) PV modules. The value can be realized by making new products after recovering and recycling this valuable material and thus contributing to the circular economy^32^. Closed-loop recycling tries to recover products and secondary materials that can be added to the original product’s supply chain. In this instance, resources and goods like silicon wafers are being salvaged and used to create fresh photovoltaic cells. When the quality of the materials isn’t good enough for the original product but may still have a lesser value and use in other industries, open-loop recycling occurs. A "cascade" of recycling can be thought of as a situation where the material has many design lifetimes and prospects for recovery in successively less valuable products while yet maintaining its utility as a product after processing.

There are mainly four thin-film advanced technologies for the manufacturing of PV modules available in the market i.e. thin-film silicon (Mono-Si & Poly-Si), amorphous silicon (a-Si), cadmium telluride (CdTe), and copper indium diselenide (CIS). Some technical features of all these PV modules are presented in Table 1.

**Table 1:** The PV module features standard technologies^33^.

PV module type	PV module efficiency at 25 °C, $\eta$(%)	Nominal operating cell temperature (°C)	Temperature coefficient for module efficiency
Mono-Si	13.0	45	0.40
Poly-Si	11.0	45	0.40
a-Si	5.0	50	0.11
CdTe	7.0	46	0.24
CIS	7.5	47	0.46

The performance of power produced from the solar PV modules is described by the term panel generation factor. It includes the solar irradiance, sunshine hour, and standard test conditions of irradiance and is intended by using the Eq. (1) as^13^,

1 $$Panel \,Generation\, Factor=\frac{Solar\, irradiance\times Sunshine \,hours}{Standard \,test \,conditions \,irradiance}.$$

The energy required from the solar PV module is the total watt-hour per day needed and it is calculated by Eq. (2) as,

2 $$Energy \,required \,from \,PV \,modules=\left(Peak \,energy\, requirement\times Energy\, lost\, in\, the \,system\right).$$

The size of the PV module is defined by the ratio of the total watt peak rating to the PV output power rating as described by Eq. (3),

3 $$PV \,module \,size=\frac{Total\, Watt\, peak \,rating}{PV output\, power\, rating},$$

where total watt peak rating is the ratio of solar PV energy required and panel generation factor as shown by Eq. (4),

4 $$Total\, Watt\, peak\, rating=\frac{Solar\,PV \,energy\, required}{Panel \,Generation\, Factor}.$$

### Solar batteries

The batteries are a buffer system for storing electrical energy and this electrical energy is utilized by the utility system when needed. Generally, the stored electrical energy is utilized during the period of off-sunshine hours, a cloudy and rainy season when solar radiations are not enough to generate the required electrical energy according to the demand. The application of batteries also depends on the type of solar PV project, for example, in the off-grid PV system, batteries are essential components because this type of system is a stand-alone system and it requires an electrical storage system to provide electrical energy during off sunshine hours at regular intervals, while in case of grid-associated solar PV system batteries are not an essential component because of grid playing the role of the distribution of electrical energy regularly^34^. There are only a few types of batteries available for application in solar PV power plants, like lead-calcium, lead-antimony, and nickel–cadmium. The nickel–cadmium batteries are particularly suitable for a wide range of temperatures. In the solar PV system, there is a fluctuation of solar radiation throughout the daytime so there is a requirement for batteries to withstand many numbers of cycles of charging and discharging throughout their life span. The lead-calcium batteries are recommended for a comparatively fewer number of cycles of operation where less than 20% discharge occurs in each cycle. While nickel–cadmium and some lead-antimony batteries are recommended for a large number of cycles where the discharge can be exceeded by 80%. The characteristics of batteries are defined by their voltage and capacity. In the case of voltage rating, it is a multiple of twelve-volt (12 V) for most cases. While the capacity of batteries is defined in terms of ampere-hour (Ah), i.e. a battery of rating of 50 Ah and 24 V will store 1200 Wh. (= 50 × 24) electricity under nominal operating conditions. The capacity of a battery in terms of ampere-hour is defined by the Eq. (5) as^13^,

5 $$CB=\frac{Daily \,Power\, Consumption\times Days\, of \,autonomy}{Battery \,Efficiency\times Depth \,of \,Discharge\times Battery \,Nominal \,Voltage}.$$

### Power conditioning unit

The power conditioning unit comprises some important electronic devices for smoothening the operations and performance of solar PV systems like battery charge controllers (BCCs), maximum power point trackers (MPPT), and rectifiers. The BCCs are adjusting the amount of current from solar panels to the batteries and, it prevents the batteries from overcharging and over-discharging. So that the performance of batteries could be better and constant throughout their life span. The MPPT is used to preserve the effective voltage of the array to maximize the output of an array. The rectifiers are battery chargers it converts the AC generated by the generator into the DC when required to charge the batteries.

### Solar inverter

The inverter is an essential electronic device used in grid-associated as well as off-grid solar PV power plants. It converts the DC output of the array or from the batteries into AC, which is the most desirable form of current in both cases of grid-associated and off-grid solar PV systems. In the case of a grid-associated system, it is designed to transport only AC while for the off-grid system; most appliances of the home are driven by DC. Here the inverter size is calculated by taking the product of energy consumed in total wattage and the factor of safety, explained by the Eq. (6) as^13^,

6 $$Inverter\, Size=\left(Total \,Wattage\,of\,the\, energy \,consumed\times factor \,of\,safety\right).$$

The grid-tied inverter coordinated with the grid on its own. The grid-tied or synchronised inverter first detects the grid's AC voltage before producing a local sine wave output with a matching voltage, amplitude, and phase. It completely ceases to function if there is no grid voltage. A phase-locked loop (PLL) control system is used to accomplish this. It is an electronic circuit that continuously modifies the frequency of an oscillator to match the frequency of an input signal. A closed-loop feedback control circuit that is phase- and frequency-sensitive is known as a phase-locked loop. A PLL is a system made up of analogue and digital components connected in a “negative feedback” topology rather than a single component.

Synchronizing the output oscillator signal with a reference signal is the PLL’s primary objective. Phase errors between the input and output frequencies are lessened using a PLL. The system is said to be “locked” when there is no phase difference between these signals. The PLL’s capacity to deliver negative feedback, or to send the output signal back to the phase detector, is also necessary for this locking operation. A PLL aids in establishing the input–output phase connection to produce the proper control voltage in addition to synchronising the output and input frequencies. As a result, it aids in a circuit's attainment of frequency and phase lock.

## Performance analysis parameters for grid-associated solar PV system

The parameters of performance analysis for grid-associated solar PV systems are described by International Electrotechnical Commission^35^. All appropriate and common parameters are discussed here as^36–41^,

### Energy produced by PV array system (E_***DC***_***)***

The entire monitored day-to-day DC power is given by Eq. (7),

7 $${E}_{DC,d}=\sum_{t=1}^{t={T}_{rp}}{V}_{DC}\times {I}_{DC}\times {T}_{r},$$

where, ${T}_{r}$ represents a time intermission of recording and ${T}_{rp}$ represents the duration of reporting.

The monthly generated DC energy is given by Eq. (8),

8 $${E}_{DC,m}=\sum_{d=1}^{N}{E}_{DC,d},$$

where *N* describes the number of operating days of a plant in a month, subscript d denotes daily value, subscript m denotes monthly value, and ${P}_{DC}={V}_{DC}\times {I}_{DC}$ is the DC power.

### Energy fed to the utility grid or output energy (E_AC_)

The whole everyday observed value of AC power production is calculated by Eq. (9) as,

9 $${E}_{AC,d}=\sum_{t=1}^{t={T}_{rp}}{V}_{AC}\times {I}_{AC}\times {T}_{r}.$$

And, AC output generated in a month is presented by Eq. (10) as,

10 $${E}_{AC,m}=\sum_{d=1}^{N}{E}_{AC,d},$$

where, ${P}_{AC}={V}_{AC}\times {I}_{AC}$ is the AC power.

### Array yield (Y_a_)

The array yield is calculated by taking the ratio of generated DC energy to the rated value of the PV power plant system^38^. The daily array yield (${Y}_{a,d}$) is presented by Eq. (11),

11 $${Y}_{a,d}=\frac{{E}_{DC,d}}{{P}_{PV, rated}}.$$

The monthly mean value of array yield (${Y}_{a,m}$) is presented by Eq. (12),

12 $${Y}_{a,m}=\left(\frac{1}{N}\right)\times \sum_{d=1}^{N}{Y}_{a,d}.$$

Array yield denotes the duration of time acquired by the PV system to operate at nominal power generation in the unit of h/day. It reflects the real task of the photovoltaic generator.

### Final yield (Y_***f***_***)***

It is described by the ratio of the final generated power to the rated PV power as detailed by the producer at the standard condition of temperature. It signifies the duration of time grabbed by the PV system to produce the final output concerning its nominal power capacity. The daily ($${Y}_{f,d})$$ and monthly $$({Y}_{f,m})$$ average final yields are given by Eq. (13) and (14) as^36^,

13 $${Y}_{f,d}={E}_{AC,d}/{P}_{PV,rated}$$

14 $${Y}_{f,m}=\left(\frac{1}{N}\right)\times \sum_{d=1}^{N}{Y}_{f,d}$$

### Reference yield (Y_r._)

It is described by the ratio of the entire in-plane solar insolation to the reference irradiance at standard temperature conditions of 1 kW/m^2^, presented by Eq. (15) as^39^,

15 $${Y}_{r,d}={T}_{r}\times \frac{{\sum }_{day}{G}_{i}}{{G}_{STC}}.$$

Further, corrected reference yield is considered by taking the modification effect of the module and ambient temperature, as presented by Eq. (16),

16 $${Y}_{cr}={Y}_{r}\left[1-{C}_{t}\left({T}_{m}-{T}_{STC}\right)\right],$$

where, ${Y}_{r}$ is the reference yield, ${C}_{t}$ is the coefficient of temperature, ${T}_{m}$ is the module temperature and ${T}_{STC}$ is the ambient temperature.

### PV module efficiency (${{\varvec{\eta}}}_{{\varvec{P}}{\varvec{V}}}$***)***

The efficiency of the PV module shows the actual generated energy through the module concerning the existing radiation. It is presented by Eq. (17) as,

17 $${\eta }_{PV}={[P}_{DC}/{(G}_{i}\times {A}_{m})]\times 100 \%,$$

where, ${P}_{DC}$ is the generated DC power, ${G}_{i}$ is the global solar irradiance and ${A}_{m}$ represents the PV module area. The monthly efficiency of the module is given by Eq. (18) as,

18 $${\eta }_{PV,m}=[{E}_{DC,m}/{(G}_{i}\times {A}_{m})]\times 100 \%,$$

where, ${E}_{DC,m}$ is the total monthly DC energy output.

### Inverter efficiency (${{\varvec{\eta}}}_{{\varvec{i}}{\varvec{n}}{\varvec{v}}}$)

The inverter efficiency can be calculated by taking a ratio of generated AC power from the inverter to the generated DC power from the PV array system. While calculating the efficiency of the inverter it is assumed that module and system efficiency is the highest, it is the transformation efficiency of DC to AC power. The instantaneous inverter efficiency is given by Eq. (19) as,

19 $${\eta }_{inv}=\left({P}_{AC}/{P}_{DC}\right)\times 100 \%.$$

The inverter efficiency can also be calculated with the help of monthly values of AC energy generated ${(E}_{AC,m})$ and DC energy generated ${(E}_{DC,m})$ by Eq. (20) as,

20 $${\eta }_{inv,m}=({E}_{AC,m}/{E}_{DC,m})\times 100 \%.$$

### System efficiency (${{\varvec{\eta}}}_{{\varvec{s}}{\varvec{y}}{\varvec{s}}}$)

The efficiency of a photovoltaic system is described by the product of the efficiency of the PV module (${\eta }_{PV})$ and efficiency of the inverter (${\eta }_{inv})$, it is presented by Eq. (21) as,

21 $${\eta }_{sys}={\eta }_{PV}\times {\eta }_{inv}.$$

### Performance ratio (PR)

The performance ratio is described by the ratio of the final yield ($${Y}_{f}$$) to the array yield ($${Y}_{a})$$, shown by Eq. (22) as,

22 $$PR={Y}_{f}/{Y}_{a}.$$

It can also be defined as a function of degradation efficiency (${\eta }_{degr}$), temperature efficiency (${\eta }_{temp})$, soiling efficiency (${\eta }_{soil})$, and inverter efficiency (${\eta }_{inv})$ as shown by Eq. (23),

23 $$PR=\left({\eta }_{degr}\times {\eta }_{temp}\times {\eta }_{soil}\times {\eta }_{inv}\right).$$

This ratio shows the actual nearness to the ideal efficiency along with the effect of losses associated with the photovoltaic system^36,38^.

### Capacity factor (CF)

It is described by the ratio of the true yearly output of energy to the amount of energy that can be delivered by the rated capacity of the PV system for 24 h. per day for one year, shown by Eq. (24) as,

24 $$CF=\frac{{Y}_{f, annual }}{(24\times 365)}=\frac{{E}_{AC,annual}}{{P}_{PV,rated}\times 8760}.$$

The CF of the solar system can also be presented by Eq. (25) as,

25 $$CF=\frac{\frac{h}{day} of\, the\, peak\, sun}{24 h/day}.$$

The CF varies in the same proportion as the variation in the final yield. It is the method to present the energy transported from an electrical power distribution system. If the system continuously supplies full-rated power, then its CF will be 1.

## Simulation methodology

Various simulation software tools are available like Photovoltaic systems (PVsyst), Hybrid optimization model for electric renewables (HOMER), System advisor model (SAM), Renewable energy technologies screen (RETScreen), PV*SOL Premium, Solar Pro, PV F-Chart, Solar Gis PV Planner, Helioscope, and Solarius PV for the analysis of solar systems. These software tools are frequently used by engineers and researchers for performance and viability analysis. And, it is observed that RETScreen has the highest compatibility with the presented research work. Even it is also driven by a large number of similar published research work on RETScreen clean energy management software. The simulation model of RETScreen is competent to compute the production of energy and the performance of financial viability analysis for photovoltaic projects like water pumping systems for small-scale applications, off-grid systems for residential applications, and grid-associated systems for large-scale applications anywhere around the globe. It is an advanced and exceptional judgment-making tool for the renewable energy field. For analyzing the performance and viability at any location around the world, environmental data of that location plays an important role. It is very difficult to collect all the environmental data at the pre-feasibility stage of any project. The RETScreen software uses NASA surface metrology and solar energy data set to overcome the problem of data collection. This environmental data set is prepared by NASA with the collaboration of RETScreen International. This data set is very much useful in case of ground-based data for the project location is not available.

**Figure 2:**
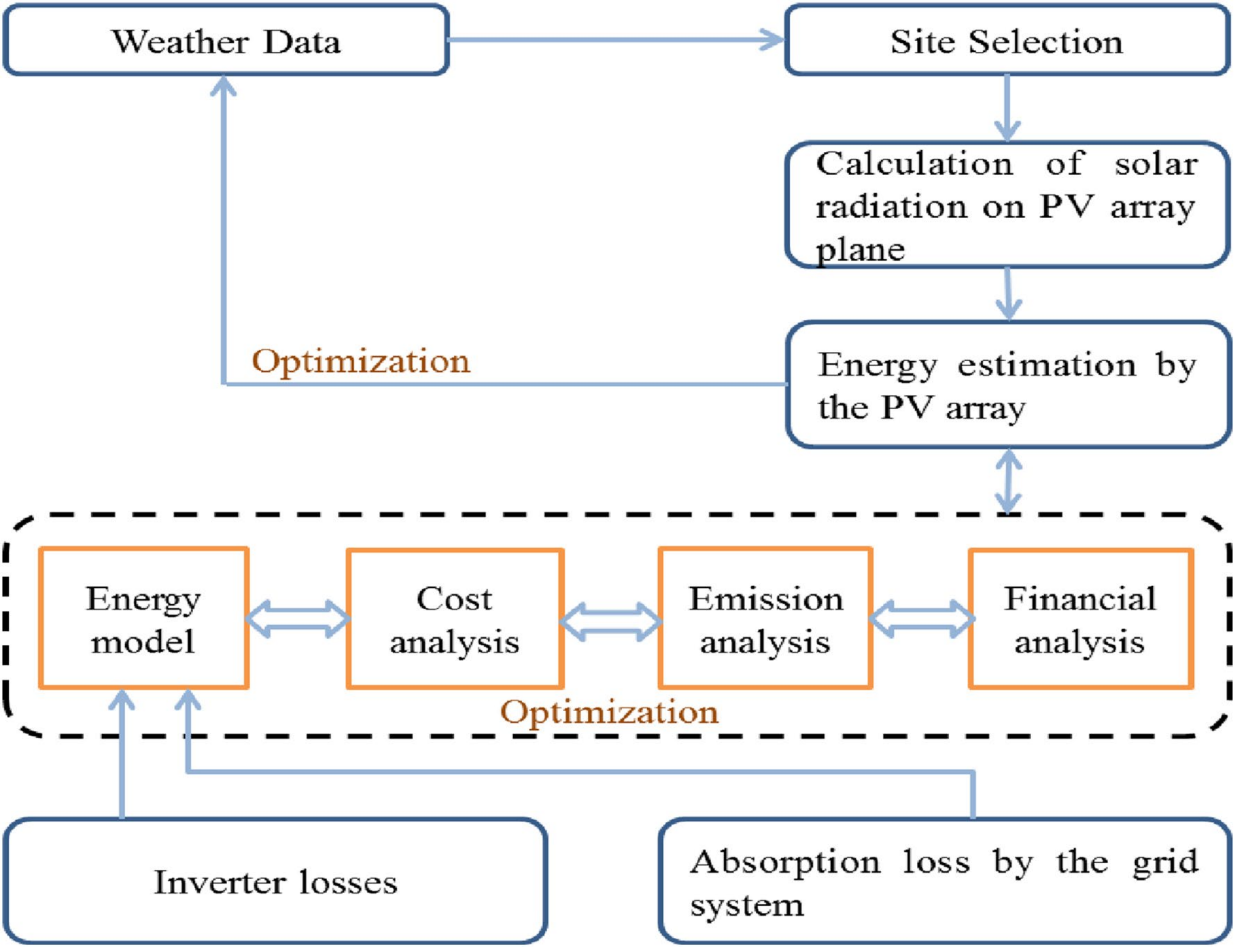
Photovoltaic energy model flowchart.

The simulation model of RETScreen contains 6 worksheets for making a simulation model of a photovoltaic project namely solar resource & system load calculation (SRSLC), energy model (EM), cost analysis (CA), financial summary and sensitivity analysis (FSSA), and greenhouse gas emission decrease analysis (GHG Analysis) in the simulation workbook file. The EM worksheet has a facility for input variables of resource assessments like solar tracking mode, slope, and azimuth; photovoltaic input variables like type, power capacity, and miscellaneous losses; inverter input variables like efficiency, capacity, and miscellaneous losses. The worksheet of SRSLC is used to recognize the type of system for the simulation model. After considering the type of system it calculates the monthly energy load. For any PV array orientation, it estimates the yearly solar radiation on the surface of a slanted array by using the monthly values of solar radiation on a horizontal surface. The CA worksheet is used to calculate the total cost of energy projects based on the RETScreen database. The worksheet of GHG analysis is used to calculate the total yearly decrease potential of greenhouse gas emission in the unit of tCO_2_/year. This worksheet also facilitates the estimation of the equivalent of GHG emission in terms of cars & light trucks that are not used, liters of gasoline not consumed, barrels of crude oil not consumed, acres or hectares of forest absorbing carbon, and tonnes of waste recycled on an annual basis. The FSSA worksheet provides the financial summary and associated risk of the proposed energy project along with the facility of input variables like escalation rate of fuel cost, rate of inflation, rate of discount, reinvestment rate, and life of a project. The EM & SRSLC worksheets should be completed first. After that, the worksheet of the CA should be completed and finally, the FSSA worksheet has to be completed. This sequence of the process could be repeated several times to achieve an optimum design for the photovoltaic project according to the use of energy and cost point view^42^. The energy model flow chart for the photovoltaic project is presented in Fig. 2. The fundamental equations for the calculation of all six worksheets have been provided in the appendix proofs (Supplementary Information).

## Description of sites and simulation parameters

**Figure 3:**
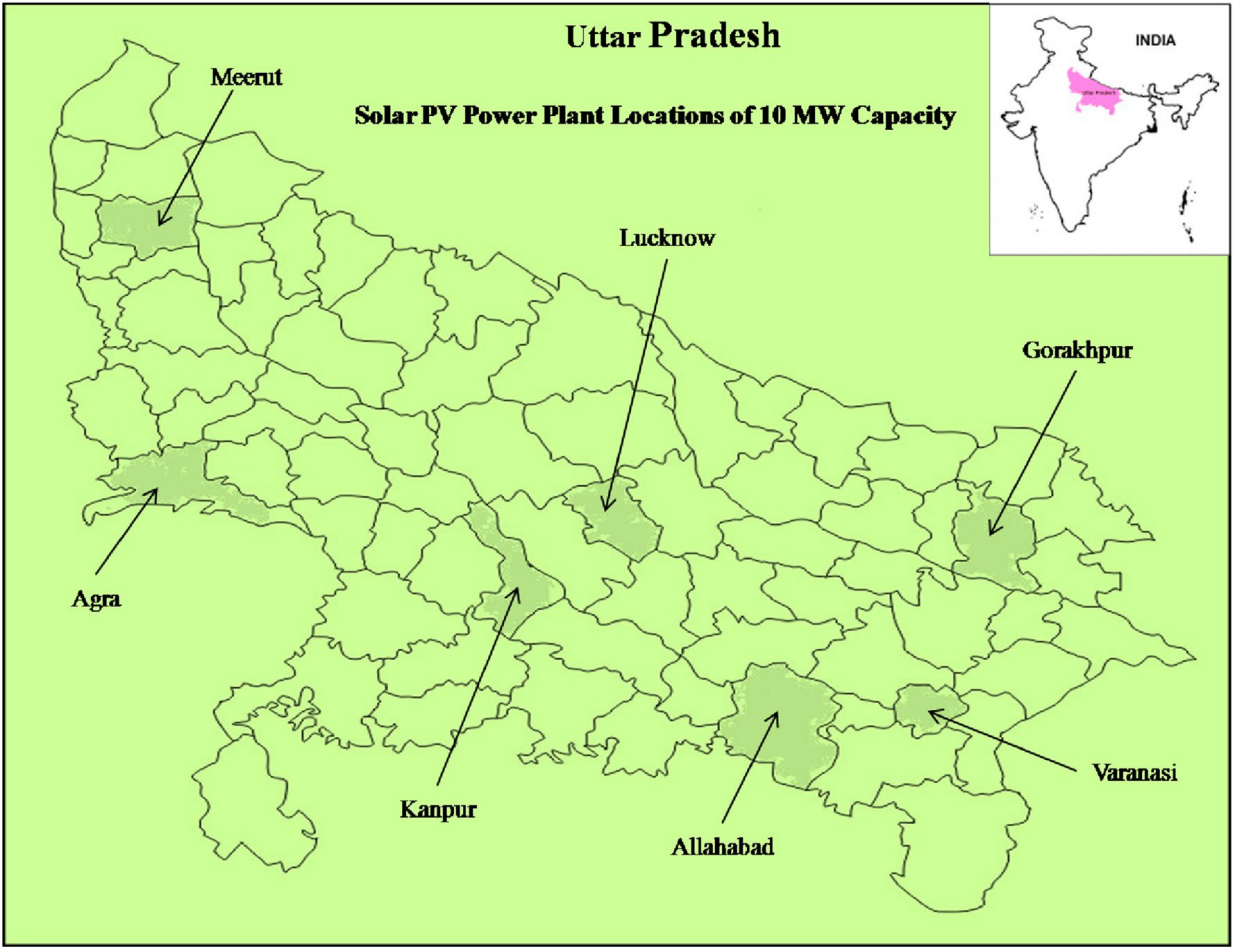
Proposed Solar PV power plant locations of UP India (https://d-maps.com/carte.php?num_car=9181&lang=en).

In the present study seven cities i.e. Lucknow, Agra, Meerut, Gorakhpur, Kanpur, Allahabad, and Varanasi with high electricity demand have been chosen for modeling of 10 MW_P_ capacity of the grid-associated solar PV power plant as shown in Fig. 3. The geographical coordinates and elevation along with key facts of selected sites are presented in Table 2.

**Table 2:** Geographical location and key facts of all the cities.

Districts	Elevation (m)	Coordinates		Area (km^2^)	Population (Million)	Mean annual PM2.5 concentration (μg/m^3^)	Annual peak energy demand (MW)
		°N	°E				
Lucknow	126	26.8	80.9	2528	2.9	83.5	1960
Agra	176	27.2	78.0	1844	1.6	91	390
Meerut	225	28.9	77.7	141.94	1.3	99.2	900
Gorakhpur	86	26.8	83.4	141	0.67	79.2	210
Kanpur	129	26.4	80.3	403	2.8	79.7	650
Allahabad	99	25.5	81.7	900	1.2	77.5	380
Varanasi	82	25.3	83.0	112.1	3.6	136	NA

NASA data & Uttar Pradesh: Uncovering solar rooftop potential in urban cities^5^.

**Figure 4:**
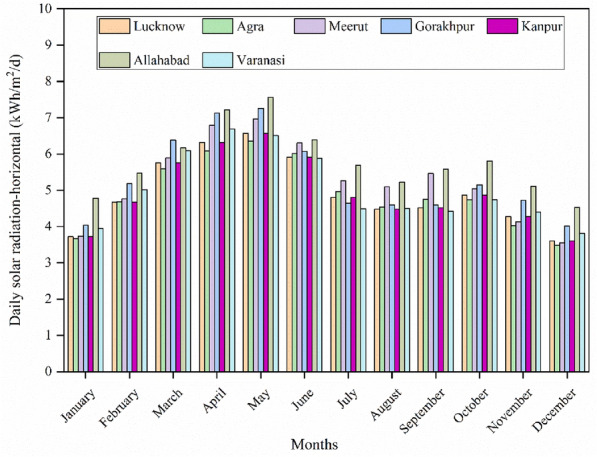
Monthly variation of solar radiation-horizontal.

The solar radiation of each city is the key parameter of energy generated at the PV array at each location of the energy project. The month-wise variation of everyday solar radiation-horizontal (kWh/m^2^/d) of each city is presented in Fig. 4.

The annual weather data of all selected cities are presented in Table 3. It includes annual mean values of air temperature, relative humidity, wind speed, earth temperature, and daily solar radiation- horizontal.

**Table 3:** Annual weather data of all the cities.

Cities	Air temperature^1^ (°C)	Relative humidity^2^ (%)	Wind speed^3^ (m/s)	Earth temperature^4^ (°C)	Daily solar radiation- horizontal^5^ (kWh/m^2^/d)
Lucknow	24.9	67.1	2.0	26.5	4.96
Agra	26.5	40.0	3.0	26.8	4.91
Meerut	25.1	61.1	2.4	25.9	5.25
Gorakhpur	26.1	48.7	2.9	26.3	5.31
Kanpur	24.9	67.1	2.0	26.5	4.96
Allahabad	25.9	59.2	3.0	26.9	5.79
Varanasi	26.4	48.4	3.0	26.8	5.04

Superscript1, 2-Ground data & 3, 4, 5- NASA data.

Among all weather data, annual daily solar radiation is an essential parameter of photovoltaic electricity generation. It has been observed from the Table 3, that the minimum value of annual daily solar radiation is 4.91 kWh/m^2^/d for the city of Agra and the maximum value of 5.79 kWh/m^2^/d for Allahabad city, and other values are well above the value of 4.5 kWh/m^2^/d which shows that enriched amount of availability of solar radiation of all selected sites.

The required input variables, like photovoltaic type, power capacity, solar tracking mode, slope, azimuth, manufacturer, model, the number of units, solar collector area, inverter capacity, fuel cost escalation rate, inflation rate, discount rate, reinvestment rate, project life, etc, for simulation of 10MW_P_ grid-associated solar PV power plant are presented in Table 4.

**Table 4:** Simulation input parameters.

Particulars	Input Values
Photovoltaic Type	Mono-Si
Power Capacity	10 MW_P_
Solar tracking mode	Fixed
Slope	Latitude
Azimuth	Zero
Manufacturer	Bosch Solar Energy
Model	Mono-Si-CC-Si M 60-NA42117-245 W
Number of units	50,000
Efficiency- Photovoltaic system	14.9%
Nominal operating cell temperature	45 °C
Temperature coefficient	0.4%
Solar collector area	67,114 m^2^
Miscellaneous losses- Photovoltaic System	15%
Inverter efficiency	95%
Inverter capacity	9000 kW
Miscellaneous losses- Inverter	1%
Fuel cost escalation rate	5%
Inflation rate	2.5%
Discount rate	12%
Reinvestment rate	9%
Project life	25 yr.

The simulation output parameters of all selected cities are summarized in Table 5. The parameters contain variables like electricity exported to the grid, electricity export revenue, SPP, EPP, pre-tax IRR-equity, pre-tax MIRR-equity, pre-tax IRR-assets, pre-tax MIRR-assets, net present value, benefit-cost (B-C) ratio, annual life cycle savings, and the gross annual GHG emission reduction of all selected cities.

**Table 5:** Simulation output parameters.

Output Parameters	Values						
	Lucknow	Agra	Meerut	Gorakhpur	Kanpur	Allahabad	Varanasi
Electricity exported to grid-annually* (MWh/Yr.)	14,319	14,083	15,329	15,378	14,277	16,686	14,381
Electricity export revenue-annually (US$/Yr.)	17,932.24	17,636.44	19,197.06	19,259.21	17,880.26	20,896.30	18,010.82
Simple payback period (Yr.)	11.1	11.3	10.3	10.3	11.2	9.4	11.1
Equity payback period (Yr.)	11.9	12.6	9.8	9.7	12.1	7.7	11.8
Pre-tax IRR—equity (%)	12	11.6	13.9	14	11.9	16.5	12.1
Pre-tax MIRR—equity (%)	10.6	10.4	11.4	11.4	10.6	12.3	10.7
Pre-tax IRR- assets (%)	3.3	3.1	4.2	4.3	3.3	5.4	3.4
Pre-tax MIRR- assets (%)	5.4	5.2	6.2	6.2	5.4	7	5.5
Net present value (US$)	78.87	− 2647.06	11,734.92	12,307.72	− 400.24	27,394.59	803.07
Benefit-cost (B-C) ratio	1	0.95	1.2	1.2	0.99	1.5	1
Annual life cycle savings (US$/Yr.)	10.06	− 337.50	1496.21	1569.23	− 51.03	3492.82	102.39
Gross annual GHG emission reduction (tCO_2_/Yr.)	16,773.4	16,496.7	17,956.5	18,014.6	16,724.8	19,545.9	16,846.9

*Standard error = simulated values have a 14% higher deviation than the actual values^21^.

## Validation of the model

**Figure 5:**
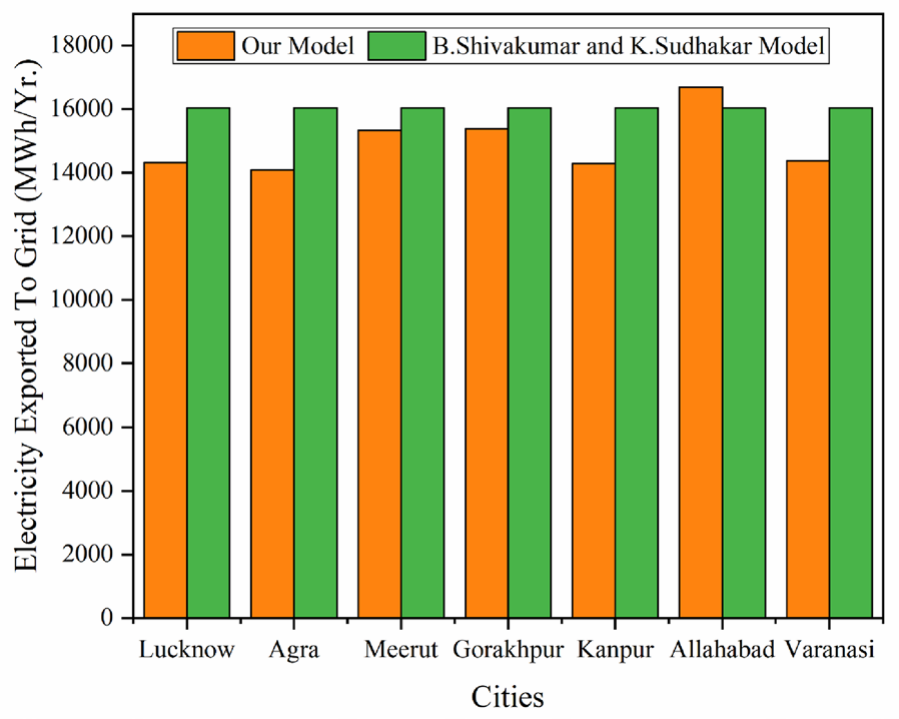
Validation of our simulated model.

The presented simulation model has been compared for electricity exported to the grid with B. Shiva Kumar and K. Sudhakar’s^29^ simulation model of a 10MW_P_ grid-connected photovoltaic power plant, in Ramagundam India. It has been found that our presented model has good agreement with it as shown in Fig. 5.

## Results and discussion

### Electricity exported and revenue generated

**Figure 6:**
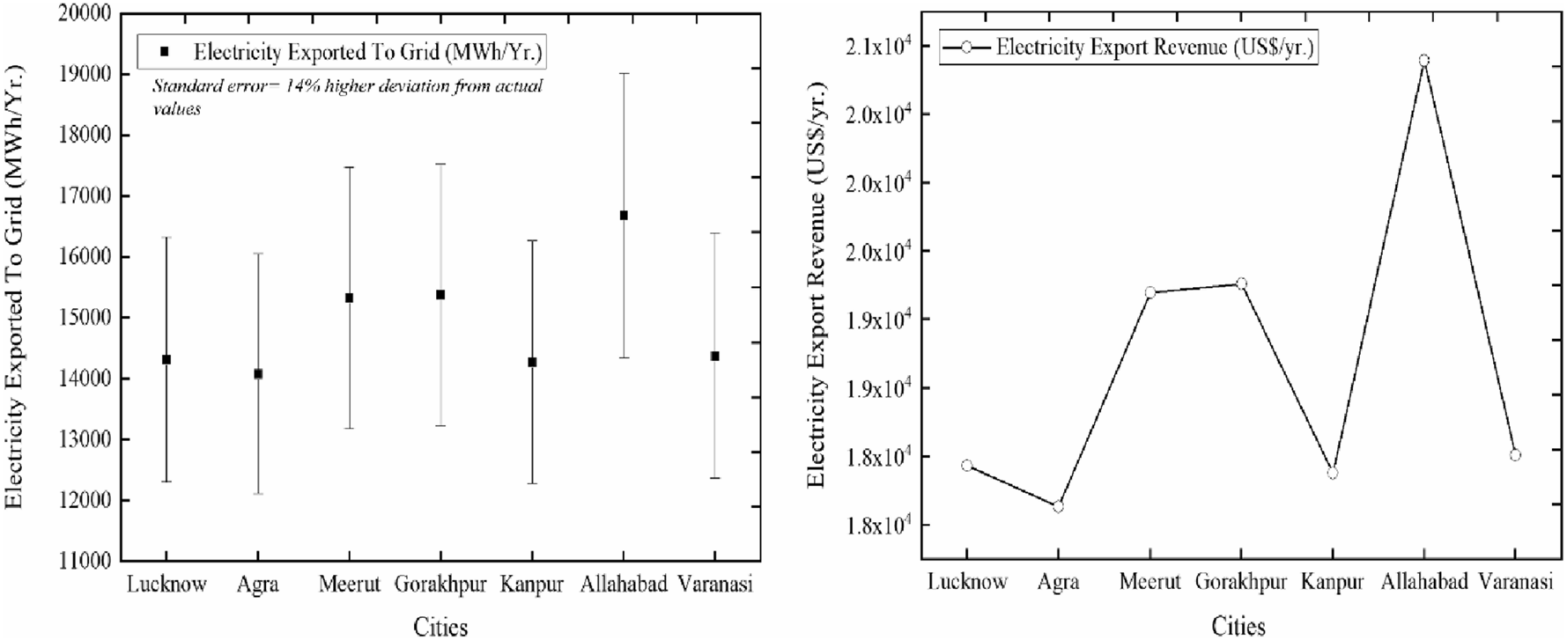
Electricity exported and revenue generated in all cities.

The electricity exported to the grid and revenue generated from it in all simulated cities is calculated here and presented in Fig. 6.

It can be evaluated from the figure that the maximum value of EEG is 16,686 MWh for the city of Allahabad along with the maximum amount of revenue generated of US$20,896.30. The minimum value of EEG is 14083MWh for the city of Agra along with the minimum value of revenue generated of US$17,636.44 among all selected cities. The second highest value of EEG is 15378MWh and the revenue generated is US$19,259.21 for the city of Gorakhpur. The other values of EEG are 14319MWh, 15329MWh, 14277MWh, and 14381MWh for the cities of Lucknow, Meerut, Kanpur, and Varanasi respectively. The corresponding values of revenue generated are US$17,932.24, US$19,197.06, US$17,880.26, and US$18,010.82 respectively for the city of Lucknow, Meerut, Kanpur, and Varanasi. Among all selected cities Allahabad is the most profitable site and Agra is the least profitable site. The second most profitable site is the city of Gorakhpur.

### Simple and equity payback period

**Figure 7:**
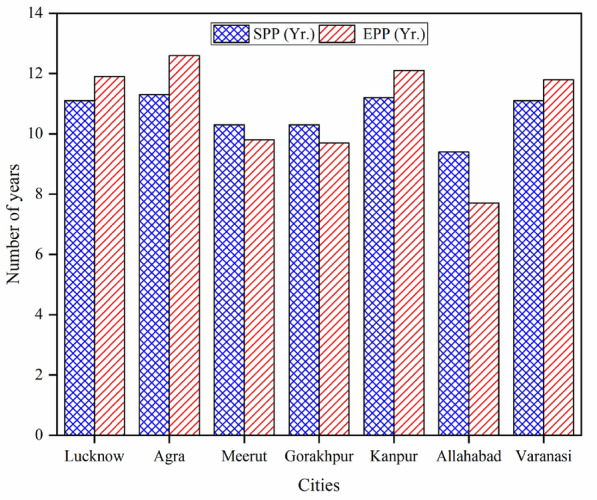
Simple and equity payback period of all cities.

The SPP is the duration of the period in which the primary cost of the proposed facility overcomes its own primary cost out of revenue and savings generated while the EPP is the length of time for the holder of the facility to get back its preliminary investment in terms of equity out of generated cash flow of the project, it is a superior time pointer of merits of the project than the SPP^43^. The SPP and EPP of each selected city are presented in Fig. 7.

It can be evaluated from the figure that the minimum value of SPP and EPP is 9.4 years and 7.7 years respectively for the city of Allahabad. The maximum value is found for the city of Agra i.e., SPP (11.3 years) & EPP (12.6 years). The second minimum value of SPP finds for two cities namely Gorakhpur and Meerut, but between these two cities, the minimum value of EPP of 9.7 years is found for Gorakhpur. The other values of SPP are 11.1 years, 11.2 years and 11.1 years corresponding to the cities of Lucknow, Kanpur, and Varanasi respectively along with the values of EPP of 11.9 years, 12.1 years and 11.8 years. According to the financial parameters of SPP and EPP, the city of Allahabad is found the most suitable site and Agra is the least profitable site. The second most profitable site is Gorakhpur.

### Pre-tax internal and modified internal rate of return on equity

**Figure 8:**
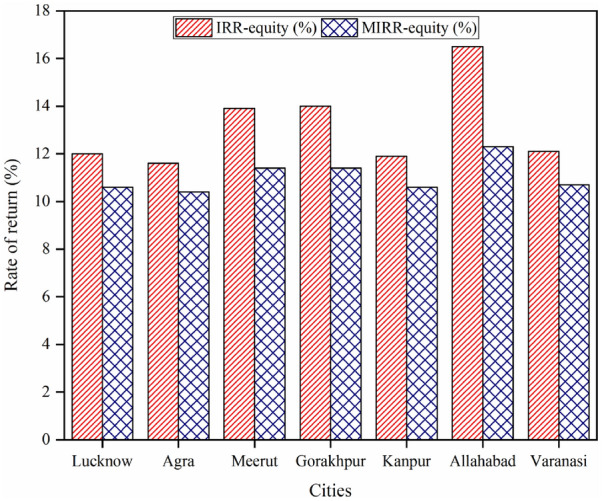
Internal and modified internal rate of return on equity of all cities.

The IRR on equity explains the accurate interest delivered by the project equity during its duration of life before income tax while the MIRR on equity denotes the profitability and cost provided by the project equity over its duration of life before income tax^44^. These two financial indicators are calculated here for each selected city and presented in Fig. 8.

It can be evaluated from the figure that Allahabad city has the highest value of the IRR and MIRR on equity i.e. 16.5 and 12.3% respectively. The second highest value of the IRR and MIRR on equity is found for the city of Gorakhpur i.e. 14 and 11.4% respectively. The minimum value of the IRR and MIRR on equity is found for the city of Agra i.e. 11.6 and 10.4% respectively. The other values of IRR on equity are 12, 13.9, 11.9 and 12.1% for the cities of Lucknow, Meerut, Kanpur, and Varanasi respectively along with the values of MIRR on equity of 10.6, 11.4, 10.6 and 10.7%. According to the IRR, and MIRR on equity, Allahabad city is the most profitable site and Agra is the least profitable site. The second most profitable site is Gorakhpur.

### Pre-tax internal and modified internal rate of return on assets

**Figure 9:**
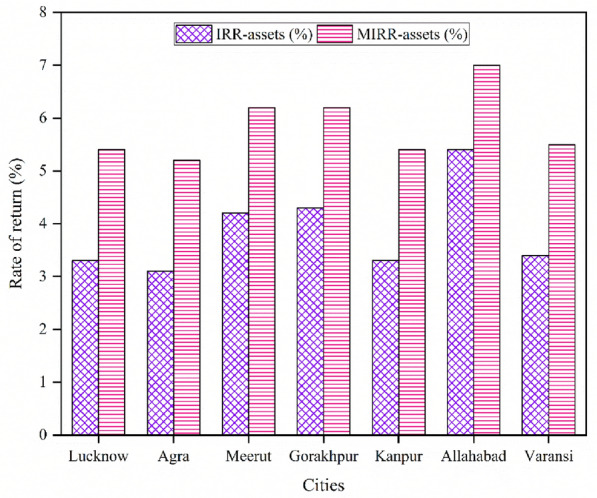
Internal and modified internal rate of return on assets of all cities.

The Pre-tax IRR on assets describes the actual interest made by assets of the project over its whole life before income tax and it is analyzed by using the pre-tax yearly cash flows & life of the project. While the Pre-tax MIRR on assets describes the actual interest made delivered by the assets of the project over its whole life before income tax. The MIRR assumes that the positive cash flows from a project are reinvested at the reinvestment rate and the negative cash flows are financed at the discount rate or a weighted average cost of capital. The IRR & MIRR on assets for each selected city is calculated here and presented in Fig. 9.

It can be evaluated from the figure that the maximum value of the IRR and MIRR on assets is found for the city of Allahabad i.e. 5.4 and 7% respectively, and minimum values are found for the city of Agra i.e. 3.1 and 5.2% respectively. The second highest values are found for Gorakhpur city i.e. 4.3 and 6.2% respectively. The other values of IRR on assets are 3.3, 4.2, 3.3 and 3.4% corresponding to the cities of Lucknow, Meerut, Kanpur, and Varanasi respectively along with the MIRR on assets of 5.4, 6.2, 5.4 and 5.5%. Based on an analysis of IRR and MIRR on assets, Allahabad is the most profitable site, Gorakhpur is the second most profitable site, and Agra is the least profitable site.

### Net present value

**Figure 10:**
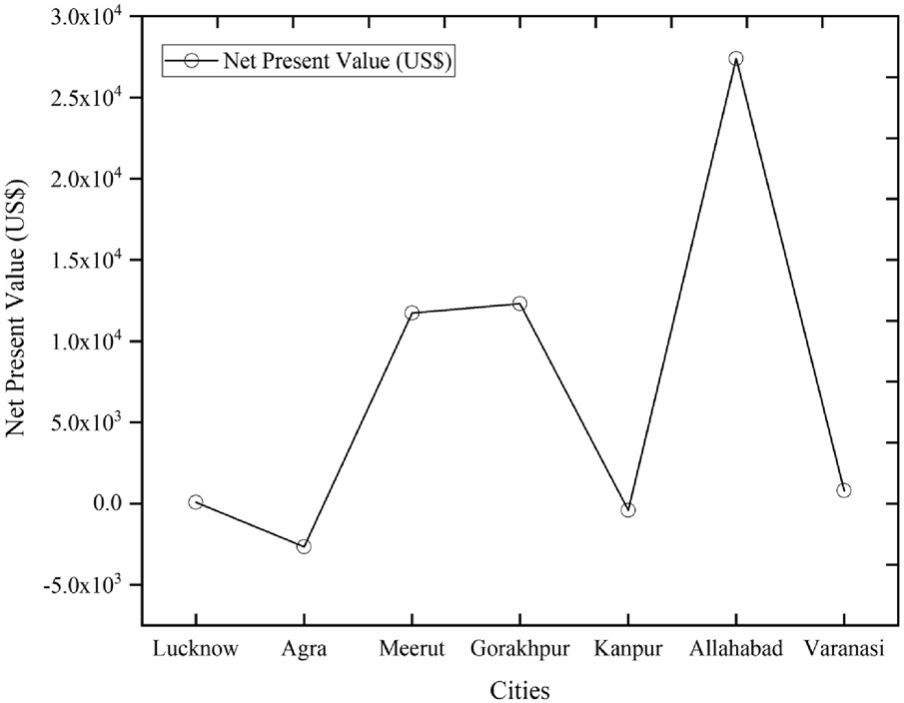
Net present value of all cities.

The NPV is the assessment of all expected cash flows of the future discounted at the discount rate in at present currency, it is associated with the IRR. Its positive values are an indication of a feasible project and its negative values are indications of a non-feasible project. The NPV of each selected city is calculated here and presented in Fig. 10.

It can be evaluated from the figure that the two cities have a negative value of NPV namely Agra and Kanpur i.e. − (US$2647.06) and − (US$400.24) respectively. These negative values indicate that these two sites are not financially feasible for solar power plant locations. The highest value of NPV is found for the city of Allahabad i.e. US$27,394.59, and the lowest value is found for the city of Lucknow i.e. US$78.87. The second highest value of NPV is found for the city of Gorakhpur i.e. US$12,307.72. The other values of NPV of Meerut and Varanasi cities are US$11,734.92 and US$803.07 respectively. The NPV analysis indicates that the most profitable site is Allahabad and the least profitable site is Lucknow city. The second most profitable site is Gorakhpur.

### Benefit-cost (B-C) ratio

**Figure 11:**
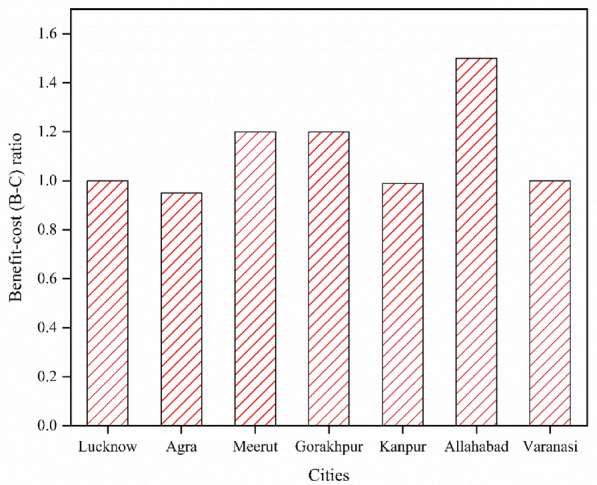
Benefit-to-cost ratio of all cities.

The net B-C ratio is the ratio of the net benefits of the project to the costs of the project. It represents the present value of annual revenue and savings as the project equity. If the ratio is greater than one then the project is profitable and similarly, if the ratio is less than one then the project is generally not profitable. The B-C ratios of all cities are calculated and presented in Fig. 11.

It can be evaluated from the figure that the B-C ratio of Allahabad city is greater among all cities i.e. 1.5, and the second highest value is found for the two cities i.e. Gorakhpur and Meerut (1.2). The two cities namely Agra and Kanpur have B-C ratios of less than one i.e. 0.95 and 0.99 respectively so these cities are not financially viable for solar power plant projects. According to the B-C ratio analysis, Allahabad is the most profitable site and the second most profitable sites are Gorakhpur and Meerut. The least profitable sites are Lucknow and Varanasi.

### Annual life cycle savings

**Figure 12:**
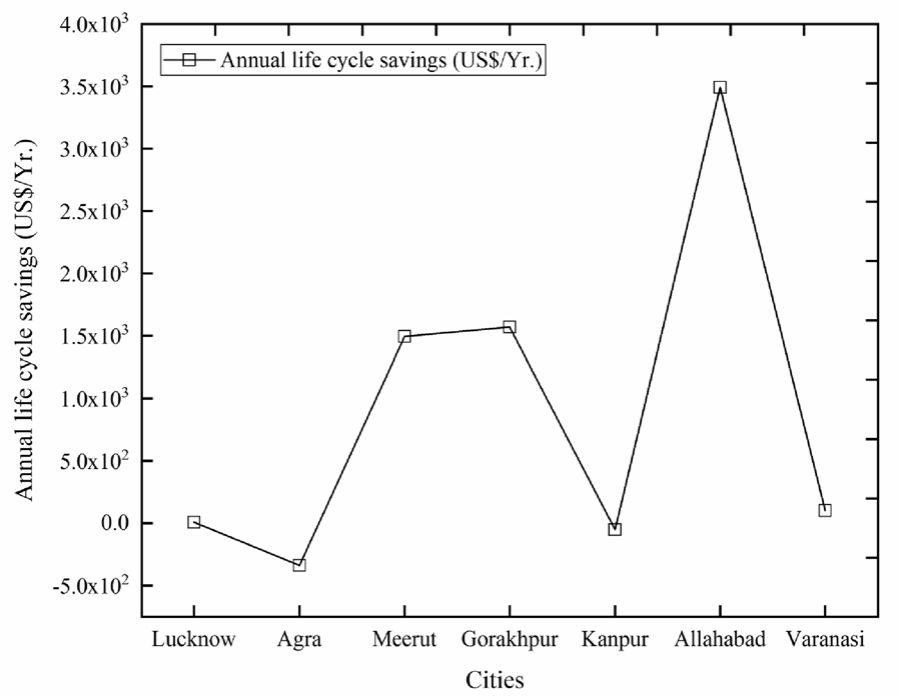
Annual life cycle savings of all cities.

The ALCS is the minimal yearly reserve having the equivalent life and NPV as the project. It is computed by considering NPV, discount rate, and project life. The ALCS at all cities is calculated here and presented in Fig. 12.

It can be evaluated from the figure that the highest value of ALCS is found for the city of Allahabad i.e. US$3492.82/year. The two cities namely Agra and Kanpur have a negative value of ALCS i.e. − (US$337.50/year.), and − (US$51.03). The negative values indicate that solar power plant project is not viable in these cities. The second highest value is found for the city of Gorakhpur i.e. US$1569.23/year. The least positive value of ALCS is found for the city of Lucknow i.e. US$10.06/year. The city of Allahabad is the most profitable site and Lucknow city is the least profitable site, along with the second most profitable site of Gorakhpur.

### Gross annual GHG emission reduction

**Figure 13:**
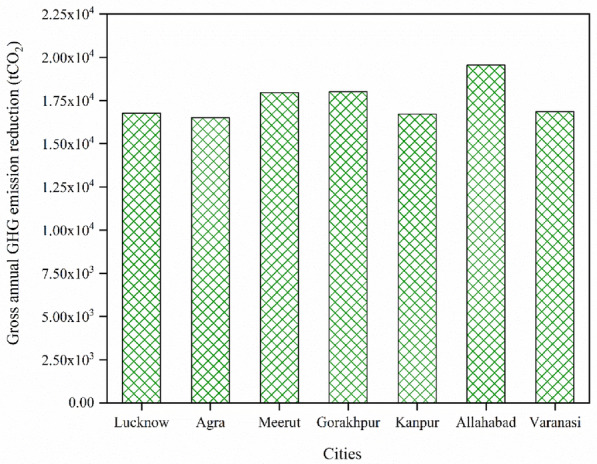
Gross yearly GHG emission reduction of all cities.

The total yearly decrease in greenhouse gas emissions can be calculated if the suggested energy project is employed. The calculation of GHG emissions is based on emissions of the base case and proposed case systems every year. The gross annual GHG emission reduction for each city is calculated and presented in Fig. 13.

It can be evaluated from the figure that the maximum amount of gross yearly GHG emission reduction is found for the city of Allahabad i.e. 19,545.9 tCO_2_/year and the minimum value is found for the city of Agra i.e. 16,496.7 tCO_2_/year. The second highest value is found for the city of Gorakhpur i.e. 18,014.6 tCO_2_/year. Based on gross yearly GHG emission reduction, the most profitable site is found in Allahabad city and the least profitable site is found in Agra city. The second most profitable site is in the city of Gorakhpur.

**Figure 14:**
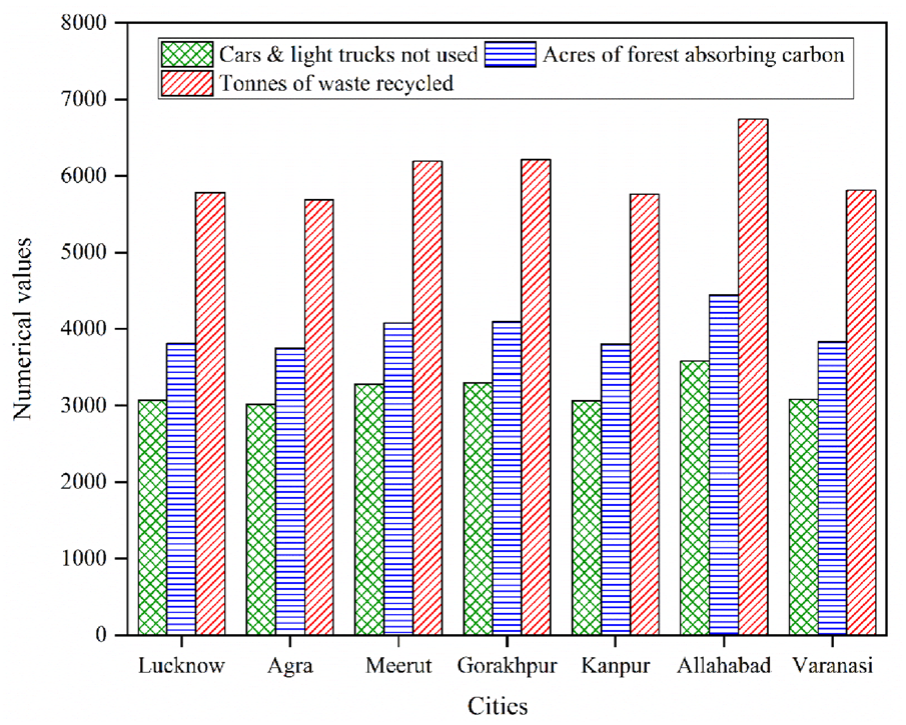
Equivalence of gross annual GHG emission reduction of all cities.

The gross annual GHG emission reduction is also expressed in more explicit forms of equivalent units of cars & light trucks not used in a year, acres of forest absorbing carbon in a year, and tonnes of waste recycled in a year. The corresponding values of all cities of gross annual GHG emissions reduction potential are presented in Fig. 14, showing that Allahabad city has the highest values among all seven cities.

## Limitations of the study

It is well-known fact that solar PV power plants required a large area of installation. It is also an important aspect whether the proposed system is grid-connected or stand-alone type. In the case of a stand-alone type, we do not bother about the distance from the grid, but in the case of a grid-connected, we should pay attention to the distance from the grid to minimize transmission losses. The location and availability of land for the proposed site also play an important role in the finalizing of sites. Generally, agricultural land is not proposed for these types of projects by the governments, due to problems in land acquisition. To overcome this issue, it is suggested that the unfertile lands, river banks, canal sides, and airport sides should be considered.

## Future outlook and recommendations

The authors believe that the presented study will be beneficial for the investigation of profitable sites for the allocation of solar PV power plants in subtropical humid climatic conditions. Even the presented simulation methodology and results will be advantageous for other similar climatic regions of the world. We also recommended that the suggested profitable sites will be further investigated through actual measured environmental ground data for fixed slope and sun-tracking systems along with sensitivity and risk analysis.

## Conclusion

It is evident that the global electricity demand is escalating at a fast rate as developing activity is increasing, which could not be fulfilled by coal-based thermal power plants alone considering the adverse environmental impacts. For this, solar PV power plants could be a credible solution, as this technology is proven advantageous around the globe. In the present study seven cities namely Lucknow, Agra, Meerut, Gorakhpur, Kanpur, Allahabad, and Varanasi in the UP state of India have been analyzed for 10 MW_P_ grid-associated solar PV power plants. The analysis is based on financial viability indicators like EEG, EER, SPP, EPP, IRR & MIRR-equity, IRR & MIRR-assets, NPV, B-C ratio, ALCS, and GHG emission reduction by the use of RETScreen Software. The key findings are as:

- The most profitable site is found in the city of Allahabad having the values of financial indicators like 16,686 MWh of EEG, US$20,896.30/year of EER, 9.4 years of SPP, 7.7 years of EPP, 19,545.9 tCO_2_ /year of GHG emission reduction, US$3492.82/year of ALCS, 1.5 B-C ratios, US$27,394.59 of NPV, 16.5% IRR-equity, 12.3% MIRR- equity, 5.4% IRR-assets, and 7% MIRR-assets.
- The second most profitable site is found in Gorakhpur city having the values of financial indicators like 15,378 MWh of EEG, US$19,259.21/year of EER, 10.3 years of SPP, 9.7 years of EPP, 18,014.6 tCO_2_/year of GHG emission reduction, US$1569.23/year of ALCS, 1.2 B-C ratios, US$12,307.72 of NPV, 14% IRR-equity, 11.4% MIRR-equity, 4.3% IRR-assets, and 6.2% MIRR-assets.
- The two cities namely Agra and Kanpur are not found financially viable for the solar power plant project of 10 MW_P_ due to being of negative values of NPV and ALCS.
- The mean values, for the remaining three cities i.e. Lucknow, Meerut, and Varanasi, of the EEG, EER, SPP, EPP, IRR & MIRR-equity, IRR & MIRR-assets, NPV, B-C ratio, and ALCS are 14,676.33 MWh/year, US$18,380.04/year, 10.83 years, 11.16 years, 12.66%, 10.9%, 3.63%, 5.7%, US$4205.62, 1.06, and US$536.22/year respectively. These values are also attractive in comparison to the most profitable site of Allahabad.
- When the mean values of EEG, EER, and SPP of the three cities (i.e. Lucknow, Meerut, and Varanasi) are compared with the most profitable site of Allahabad (as reference value) then it is found that EEG and EER are 12.04% less and SPP is higher by 15.21%. So, these values indicate that these three cities could be considered feasible sites but not profitable sites.
- The GHG emission reduction potential for the whole project life of 25 years for the most profitable site of Allahabad is 488,647.5 tCO_2_ and for the second most profitable site of Gorakhpur is 450,365 tCO_2_. And the mean value of the remaining three cities (i.e. Lucknow, Meerut, and Varanasi) is 429,806.66 tCO_2_.
- The equivalence of annual GHG emission reduction of the most profitable site of Allahabad (i.e. 19,545.9 tCO_2_) in terms of cars & light trucks not used in a year, acres of forest absorbing carbon in a year, and tonnes of waste recycled in a year are as 3579.8, 4442.3, and 6740 respectively. These equivalences of values are of great interest for understanding the importance of the installation of solar PV power plants.

The major findings could be the great interest in understanding the economic and environmental benefits of establishing solar PV power plants, not even for the humid subtropical climate of UP India but also in similar environmental conditions like Nepal, Bhutan, Pakistan, Bangladesh, Sri Lanka, and the Philippines, etc. Even the study is throwing light on creating a roadmap for investment in solar PV power plants in such types of developing countries. So, the impact of global warming should decrease to the desired level (Supplementary Information).

## Supplementary Information

Supplementary Information.

## Data Availability

The datasets used and/or analyzed during the current study are available from the corresponding author Dr. Hitesh Panchal on reasonable request.
