# Supplementary material for: Performance evaluation and financial viability analysis of grid associated 10 MWP solar photovoltaic power plant at UP India
Source: Sci Rep. 2022 Dec 26;12:22380. doi: 10.1038/s41598-022-26817-4 (PMC9792540; doi:10.1038/s41598-022-26817-4)
Supplement: Supplementary file 1 — Supplementary Information. [file 41598_2022_26817_MOESM1_ESM.docx]

**Appendix: proofs**

The fundamental equations for the calculation of all six worksheets are based on the following equations (Natural Resources Canada, 2005; IEC Standard 61724, 1998),

**Internal rate of return (IRR)**

The IRR is the concession rate and it is calculated by considering the zero net present value of the project. It is computed by the equation for IRR,

$$0=\sum_{n=0}^{N} \frac{C_{n}}{{(1+IRR)}^{n}}$$

Where N is the value in years of project life, and $C_{n}$ is the cash flow for the n^th^ year.

**Simple payback period (SPP)**

The SPP is the duration of time in years in which cash flow will be just equal to the total investment of the project. It is calculated as,

$$SP=\frac{C-IG}{\left( C_{ener}+C_{capa}+C_{RE}+C_{GHG} \right)-(C_{O\&M}+C_{fuel})}$$

Where C denotes the entire primary project cost, IG describes the value of grants and incentives, $C_{ener}$ defines the yearly income or energy savings, $C_{capa}$ denotes the yearly capacity savings or income, $C_{RE}$ denotes the yearly credit income of renewable energy production, $C_{GHG}$ denotes the income of GHG reduction, $C_{O\&M}$ describes the cost of yearly operation and maintenance of the project and $C_{fuel}$ is the yearly cost of fuel or electricity.

**Equity payback period (EPP)**

The EPP is the duration of time in which cash flow will become positive in the first year of the cumulative cash flow of the project. It is computed as for $N_{PCF}$,

$$0=\sum_{n=0}^{N_{PCF}} \tilde{C}_{n}$$

Where, $\tilde{C}_{n}$ is the cash flow for the n^th^ year after the application of tax.

**Net present value (NPV)**

The NPV is the total upcoming cash flows reduced at the concession rate in the present day’s currency. It is considered by discounting all cash flows as given in the following formula,

$$NPV=\sum_{n=0}^{N} \frac{\tilde{C}_{n}}{{(1+r)}^{n}}$$

Where r is the concession rate and n is the year.

**Annual life cycle savings (ALCS)**

The ALCS is the minimal annual reserve having an identical life and net present value as of the project. It is presented by using the following formula,

$$ALCS=\frac{NPV}{\frac{1}{r}(1-\frac{1}{\left( 1+r \right)^{N}})}$$

Where N is the end year of the project life.

**Benefit-cost (B-C) ratio**

The B-C ratio is an appearance of the comparative cost-effectiveness of the project. It is computed as a ratio of the current value of yearly revenues less yearly costs of the project equity as,

$$B-C=\frac{NPV+\left( 1-f_{d} \right)C}{\left( 1-f_{d} \right)C}$$

Where, $f_{d}$ is the debt ratio.

**Energy production cost (**$\mathbf{C}_{\mathbf{Prod}}\mathbf{)}$

The cost of energy production is the eluded cost of energy that brings the net present value to zero. The energy production cost ($C_{Prod})$ is thus calculated by solving for

$$0=\sum_{n=0}^{N} \frac{\tilde{C}_{n}}{{(1+r)}^{n}}$$

Where,$\tilde{C}_{n}=C_{n}-T_{n}$

$$C_{n}=C_{in,n}-C_{out,n}$$

$C_{in,n}=C_{prod}{(1+r_{e})}^{n}+C_{capa}{(1+r_{i})}^{n}+C_{RE}{(1+r_{RE})}^{n}+C_{GHG}{(1+r_{GHG})}^{n}$.

**Cost of GHG emission reduction**

The cost of GHG emission reduction (CGR) denotes the minimal cost to be acquired for each tonne of GHG evaded. It is presented as,

$$CGR=-\frac{ALCS}{\Delta_{GHG}}$$

Where, $\Delta_{GHG}$ is the yearly GHG emission decrease, computed from the GHG worksheet analysis.
